# Theoretical prediction of the electronic structure, optical properties and photocatalytic performance of type-I SiS/GeC and type-II SiS/ZnO heterostructures

**DOI:** 10.1039/d3ra01061a

**Published:** 2023-03-07

**Authors:** S. S. Ullah, H. U. Din, Sheraz Ahmad, Q. Alam, S. Sardar, B. Amin, M. Farooq, Cuong Q. Nguyen, Chuong V. Nguyen

**Affiliations:** a Department of Physics, Hazara University Mansehra KP Pakistan farooq@hu.edu.pk; b Computational Science Research Center, Korea Institute of Science and Technology (KIST) Seoul 02792 Republic of Korea 025264@kist.re.kr; c Department of Physics, Bacha Khan University Charsadda KP Pakistan haleem.uddin@yahoo.com; d School of Materials Science and Engineering, Institute of New Energy Material Chemistry, Nankai University Tianjin 300350 P. R. China; e Department of Physics, Abbottabad University of Science & Technology Havelian Abbottabad KP Pakistan; f Institute of Research and Development, Duy Tan University Da Nang 550000 Vietnam nguyenquangcuong3@duytan.edu.vn; g Faculty of Natural Sciences, Duy Tan University Da Nang 550000 Vietnam; h Department of Materials Science and Engineering, Le Quy Don Technical University Hanoi Vietnam

## Abstract

Nowadays, it would be ideal to develop high-performance photovoltaic devices as well as highly efficient photocatalysts for the production of hydrogen *via* photocatalytic water splitting, which is a feasible and sustainable energy source for addressing the challenges related to environmental pollution and a shortage of energy. In this work, we employ first-principles calculations to investigate the electronic structure, optical properties and photocatalytic performance of novel SiS/GeC and SiS/ZnO heterostructures. Our results indicate that both the SiS/GeC and SiS/ZnO heterostructures are structurally and thermodynamically stable at room temperature, suggesting that they are promising materials for experimental implementation. The formation of SiS/GeC and SiS/ZnO heterostructures gives rise to reduction of the band gaps as compared to the constituent monolayers, enhancing the optical absorption. Furthermore, the SiS/GeC heterostructure possesses a type-I straddling gap with a direct band gap, while the SiS/ZnO heterostructure forms a type-II band alignment with indirect band gap. Moreover, a red-shift (blue-shift) has been observed in SiS/GeC (SiS/ZnO) heterostructures as compared with the constituent monolayers, enhancing the efficient separation of photogenerated electron–hole pairs, thereby making them promising candidates for optoelectronic applications and solar energy conversion. More interestingly, significant charge transfers at the interfaces of SiS–ZnO heterostructures, have improved the adsorption of H, and the Gibbs free energy Δ_H*_ becomes close to zero, which is optimal for the hydrogen evolution reaction to produce hydrogen. The findings pave the path for the practical realization of these heterostructures for potential applications in photovoltaics and photocatalysis of water splitting.

## Abstract 2

First-principles calculations were employed to investigate the electronic structure, optical properties and photocatalytic performance of novel SiS/GeC and SiS/ZnO heterostructures.

## Graphical abstract

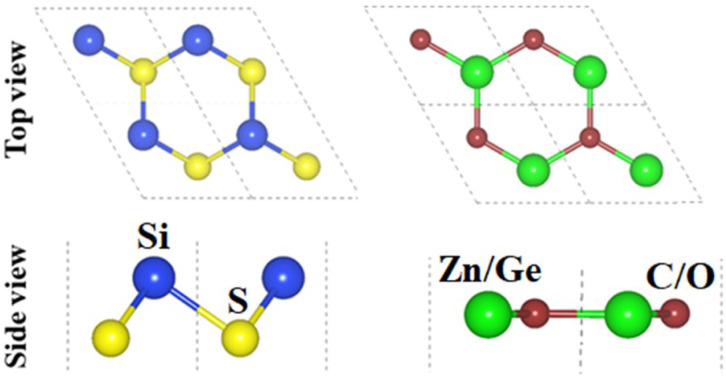

## 1 Introduction

Recently, technologies that convert solar energy into useable forms, such as hydrogen generation from the photocatalytic splitting of water and using solar cells to generate electricity, have great potential for addressing the current energy crisis and environmental challenges.^1–3^ The pairs of electrons and holes are created by photons when a semiconductor is illuminated by sunlight. The excited electrons cause the hydrogen reduction reaction to produce H_2_, while excited holes participate in oxidation to produce O_2_. However, a photocatalytic water splitting reaction is only facilitated by a semiconductor, when these two conditions are fulfilled: (i) the conduction band minimum (CBM) should have energy greater than the reduction potential for H^+^/H_2_ (−4.44 eV) and (ii) the valence band maximum (VBM) should be lower than the oxidation potential for O_2_/H_2_O (−5.67 eV).^4^ Accordingly, a main feature of photocatalytic decomposition of water is to explore a semiconductor for making photo-electrode, which is capable to absorb sunlight and driving water decomposition reaction. Numerous novel photocatalyst materials have been explored when an initial report on photocatalytic water splitting by using TiO_2_.^5^ Besides, various studies on nanoparticles of TiO_2_ have revealed an improvement in photocatalytic activities.^6–9^ Therefore, there is still challenging to discover novel materials for photocatalysis.

Nowadays, two-dimensional (2D) materials are considered as potential platform to develop photovoltaic technologies since the initial successful exfoliation of graphene^10^ owing to their intriguing features, such as tunable bandgaps, robust absorption in optical, region and higher carrier mobility.^11–14^ It is interest to create a multifunctional material that can successfully carry out the entire water splitting reaction and demonstrate prospective application in photovoltaic cells in the interest of preparation simplicity and cost reduction. However, there are still many complications in rationally designing a material with the aforementioned tri-functionality. Interestingly, the stacking of two different atomically thin 2D layered materials *via* weak van der Waals (vdW) forces has been found to have significant potential for enhanced photocatalytic applications.^15,16^ 2D vdW heterostructures offer the greatest surface area of contact between photogenerated charge carriers and water, whereas reducing the distance for migration of photogenerated electrons and holes, decreasing the chances of recombination of electrons–holes and striving to improve catalytic performance. Furthermore, an enhanced electron–electron correlation due to quantum confinement effect in these materials causing to increase the binding energy of exciton, thereby extending the lifetime of photogenerated charge carriers. In addition, 2D vdW heterostructures generate a built-in dipole moment, enhancing the kinetic overpotential of oxygen and hydrogen evolution reactions and preventing the photogenerated electron–hole recombination. For academic and commercial purposes, it is necessary to create and explore innovative, high-efficiency 2D vdW heterojunction catalysts. There is no doubt about the fact that modifying the electronic structures of 2D materials by vertically stacking different two-dimensional materials to create vdW heterostructures will result in novel and intriguing features. Especially, for the purpose of light-harvesting, type-II vdW heterostructures with staggered band-alignment can expedite the spatial separation of photogenerated electron–hole pairs and reduce their recombination. Up to date, a wide variety of type-II vdW heterostructures have been explored and exhibited promising approach for applications in photocatalytic splitting of water or photovoltaic devices.^17–20^

The Hydrogen Evolution Reaction (HER) is essential for creating hydrogen from water as a renewable energy source. Due to the wide variety of 2D material combinations that can be used, two-dimensional heterostructures in particular are one of the most promising HER approaches. In fact, earlier theoretical and experimental findings have shown that the construction of 2D heterostructures can enhance performance of HER.^21^ Currently, ZnO has received interest from the research community, and numerous studies have been conducted to synthesize ZnO layers and create its heterostructures with various 2D materials based on weak van der Waals forces.^22–24^ 2D ZnO exhibits good chemical stability, tunable electronic characteristics, and enhanced optical absorption when combined with other elemental 2D materials, including stanene,^24^ graphene,^25^ phosphorus^26,27^ and MoS_2_,^28^ thereby, making these materials promising for applications in electronic and optoelectronic devices. Additionally, graphene-like GeC monolayer has been an important 2D material in several applications. GeC monolayer has experimentally fabricated from GeC films *via* chemical vapour deposition (CVD) method.^29^ Due to its high stability and appropriate bandgap of 2.20 eV, it is a promising option for applications in a number of semiconducting devices.^30^ g-GeC possesses tunable morphological features and a honeycomb-like plain structure, just like graphene. GeC has been employed as a core material in several solar cells and a few light emitting diodes, mostly of blue or ultraviolet color.^31,32^ Furthermore, it has been demonstrated that ZnO/GeC heterostructure has direct band gap type-II band alignment and exhibited its significant potential to act as a photocatalyst with enhanced activity for processing under visible light, which lead it to be used as promising candidate for optoelectronic and photocatalytic water splitting.^33^ Theoretical study of 2D SiS based vdW heterostructures, including SiS/SiC, SiS/P and GaN/SiS demonstrated that they have potential to be used for visible light photocatalysis and optoelectronics.^34,35^ Since the SiS, GeC and ZnO monolayer are semiconductors and possess an identical structure having nearly identical lattice parameters of 3.29, 3.26 and 3.28 Å, respectively. 2D vdW heterostructure stacked by SiS monolayer with GeC and ZnO monolayers would be best photocatalysts for photocatalytic splitting of water and solar cell applications. Therefore, in this work, we perform first principles calculations to prove that SiS/g-GeC and SiS/g-ZnO vdW heterostructures are dynamically table, capable to absorb visible and ultra-violet light, suitable positions of energy band edges and complete separation of electrons and holes, as well as an intrinsic electrical field. These properties indicate that these vdW heterostructures are promising candidates for photocatalytic water splitting and photovoltaic cells to get clean renewable energy.

## 2 Computational methods

The first-principles calculations are performed using density functional theory encoded in Vienna *ab initio* simulation package (VASP)^36^ through the projected augmented wave (PAW) pseudo-potentials technique.^37^ To find the exchange-correlation energy, Perdew–Burke–Ernzerhof (PBE) type of generalised gradient approximation (GGA)^38,39^ was used in atomic structure optimization. For geometric optimization the energy and forces are set to converge at 10^6^ eV and 0.01 eV Å^−1^, respectively. To sample the Brillouin zone, a 9 × 9 × 1 *Γ*-centered Monkhorst–Packk mesh is taken for optimization and electronic structure calculations. These calculations are performed using a plane wave cut-off energy of 500 eV. We used semi empirical dispersion correction DFT-D3 approach for the long range vdW interaction in order to more accurately analyze the weak vdW forces in the layered materials.^40^ In order to prevent the interaction between adjacent layers caused by periodicity, a vacuum space 20 Å thickness is created between layers. The PBE functional determine precisely the physical characteristics of materials, however it underestimates band gap, hence the HSE06 (Heyd–Scuseria–Ernzerhof) hybrid functional^41^ was employed to more precisely determine the electronic characteristic. To find thermal stability of SiS/GeC and SiS/ZnO heterostructures, we used *Ab initio* molecular dynamics (AIMD) simulations. The Gibbs free energy is evaluated by using equation:

1 Δ*G*_H*_ = Δ*E*_H*_ + Δ*E*_ZPE_ − *T*Δ*S*

where Δ*E*_H*_, Δ*E*_ZPE_ and Δ*S* denote the adsorption energy of hydrogen, zero-point energy variation and variation in entropy during the process of H adsorption, respectively. Δ*E*_H*_ represent the difference in energy before and after H adsorption and is given by:2
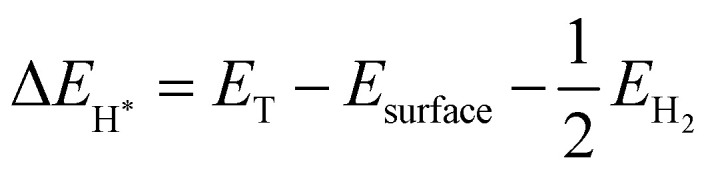


The vibrational frequency calculations give values of Δ*E*_ZPE_ for H* and H_2_. Δ*S* is nearly equal to −1/2*S*_H_2__.

## 3 Results and discussion

**Fig. 1:**
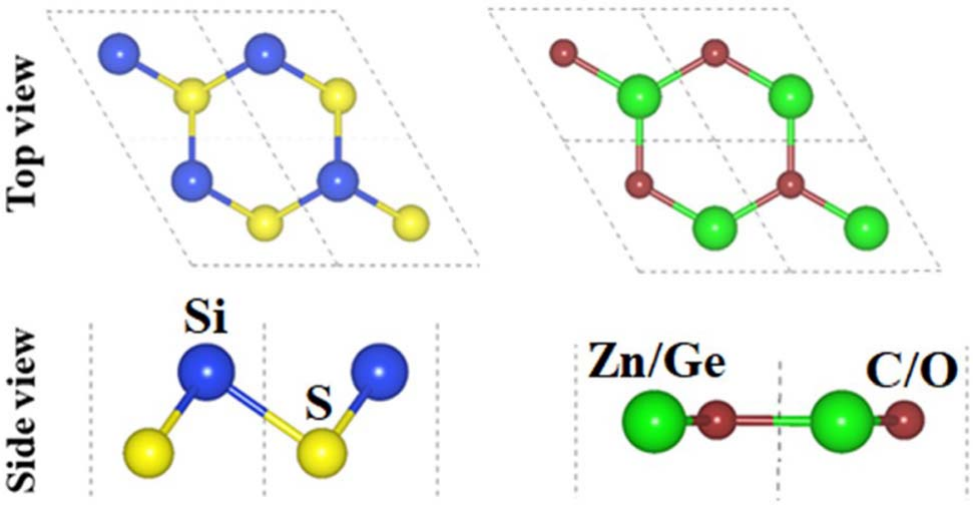
Top and side views of the atomic structures of (a) SiS and (b) GeC (ZnO) monolayer.

**Fig. 2:**
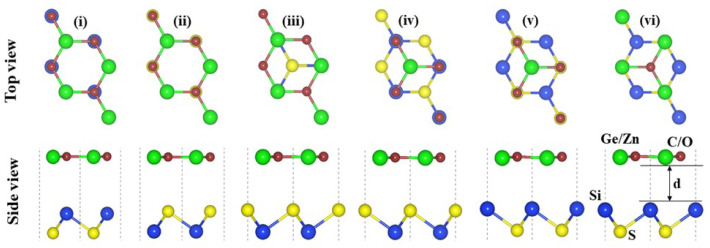
Top and side views of the atomic structures of six different possible stacking configurations for SiS/GeC (SiS/ZnO) heterostructure.

The crystal structures of SiS, GeC and ZnO monolayers are illustrated in Fig. 1. One can find that SiS monolayer exhibits buckled honeycomb structures, in which Si and S atoms are covalently bonded with each other. While g-GeC and g-ZnO monolayers have honeycomb planar structures connected by Ge/Zn and C/O atoms, respectively. Our calculations show that the SiS, g-GeC and g-ZnO monolayers have optimized lattice parameters of 3.29, 3.26 and 3.28 Å, respectively, which are in accordance with their experimental values and the previous theoretical results.^42–44^ Interestingly, the SiS and g-GeC monolayers have a lattice mismatch of about 0.92%, while such mismatch for SiS and ZnO monolayers is about 0.3%, which ensures that stacking of these materials is experimentally feasible. In addition, similar trend has also observed in other studies.^42–44^ Based on the positions of atoms contacted relative to one another of different monolayers, we take into account six different stacking configurations of the considered SiS/g-GeC and SiS/ZnO heterostructures, as illustrated in Fig. 2. In order to determine the most probable configurations of SiS/g-GeC and SiS/ZnO heterostructures, we further evaluate their binding energies (*E*_b_) and interlayer distances (*d*). The binding energy at the interface of SiS and g-GeC (ZnO) can be obtained as:

3 *E*_b_ = *E*_H_ − *E*_M1_ − *E*_M2_

**Fig. 3:**
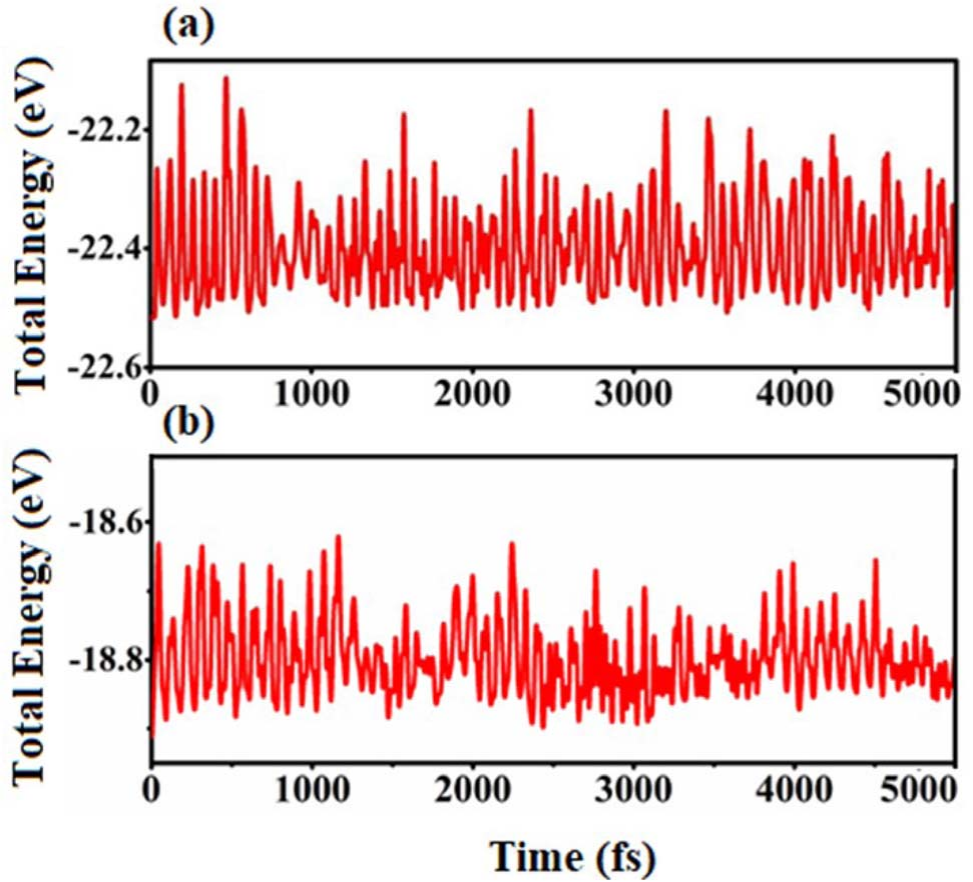
AIMD simulations of the fluctuation of total energy of (a) SiS/GeC and (b) SiS/ZnO heterostructures as a function of time steps at room temperature of 300 K.

Here, *E*_H_, *E*_M1_ and *E*_M2_ represent the total energies of SiS/g-GeC (SiS/ZnO) heterostructures, isolated SiS and GeC (ZnO) monolayers, respectively. The interlayer spacing and binding energy for both the SiS/GeC and SiS/ZnO heterostructures are listed in Table 1. One can find that the most stable pattern of both the SiS/GeC and SiS/ZnO heterostructures is stacking “v” due to the lowest binding energy and smallest interlayer distance compared to others. It is notable that stability of heterostructure can increase with decreasing binding energy. Consequently, we consider the most stable configuration of SiS/GeC and SiS/ZnO heterostructures for the subsequent calculation. A further demonstration of the thermal stability of SiS/GeC and SiS/ZnO heterostructures is provided by the AIMD calculations, as depicted in Fig. 3. We can find that the fluctuation of the total energy of both the SiS/GeC and SiS/ZnO heterostructures are small. As the time span increases, the energy and temperature exhibit a minor oscillation, without undergoing any substantial structural change. Thus, both the SiS/GeC and SiS/ZnO heterostructures are thermodynamically stable at room temperature.

**Table 1:** Calculated interlayer spacing (*d*, Å) and binding energy (*E*_b_, eV) of the SiS/GeC and SiS/ZnO heterostructure

Configurations	SiS/GeC		SiS/ZnO	
	*d*, Å	*E*_b_, eV	*d*, Å	*E*_b_, eV
Stacking (i)	3.87	−0.09	3.36	−0.12
Stacking (ii)	3.58	−0.11	3.33	−0.11
Stacking (iii)	3.23	−0.16	2.93	−0.17
Stacking (iv)	3.25	−0.15	2.96	−0.17
Stacking (v)	**3.15**	**−0.21**	**2.87**	**−0.23**
Stacking (vi)	3.20	−0.18	2.90	−0.21

**Fig. 4:**
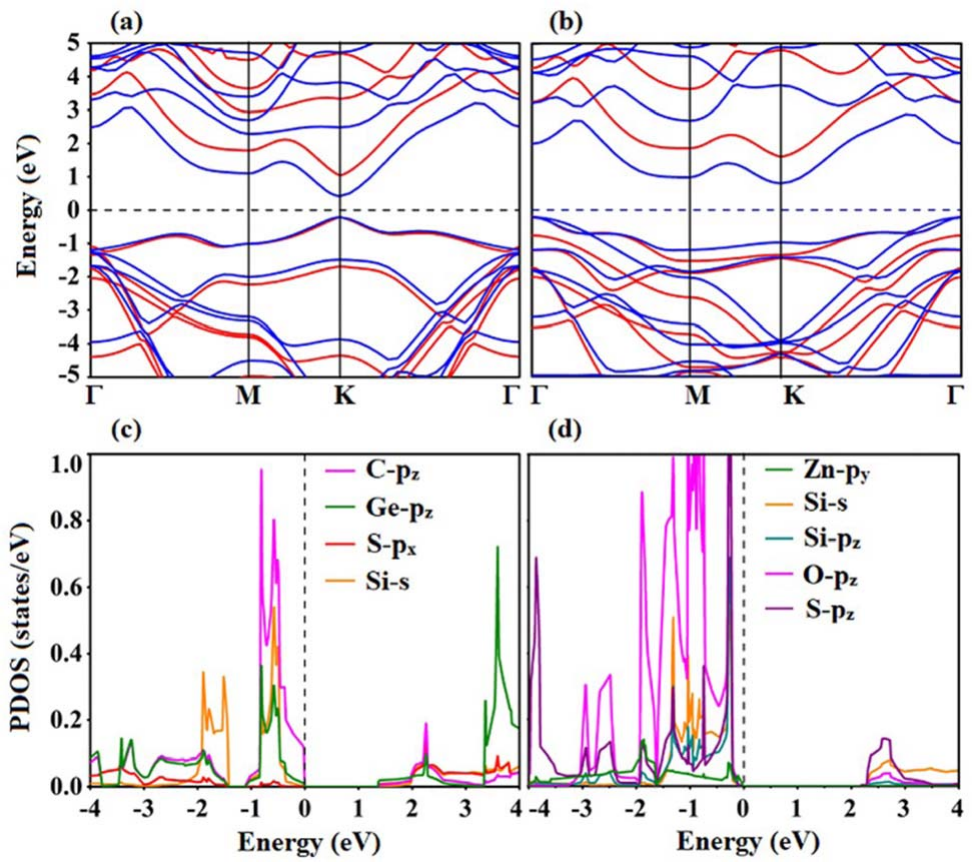
Calculated band structures of (a) SiS/GeC and (b) SiS/ZnO heterostructures obtained by PBE (blue lines) and HSE06 functional (red lines). Projected density of states (PDOS) of all atoms in the (c) SiS/GeC and (d) SiS/ZnO heterostructures. The Fermi level is marked by dashed black line and is set to be zero.

Calculations of the electronic band structures of both heterostructures provide a detailed understanding of the electronic characteristics. To better explore the electronic characteristics of SiS/GeC and SiS/ZnO heterostructures, we further illustrate their band structures by employing PBE and HSE06 functional, as depicted in Fig. 4. One can see that the SiS/GeC heterostructure exhibits a direct band gap semiconductor, while the SiS/ZnO heterostructure possesses an indirect one. Both the VBM of CBM of SiS/GeC heterostructure are located at the *K* point, as depicted in Fig. 4(a). Whereas, the VBM of the SiS/ZnO heterostructure is located at the *Γ* point and the CBM lies at the *K* point, as depicted in Fig. 4(b). The calculated band gaps of SiS/GeC and SiS/ZnO heterostructure obtained by PBE (HSE06) functional are 0.67 (1.42) and 1.10 (1.98) eV, respectively. It can be seen that these band gaps are still smaller than those of the constituent SiS, GeC and ZnO monolayers. Our calculations show that the calculated band gaps for SiS, GeC and ZnO monolayers obtained by PBE (HSE06) functional are 2.18 (2.97), 2.11 (2.91) and 1.65 (3.12) eV, respectively. The reduction in the band gaps of heterostructures indicates that the formation of SiS/GeC and SiS/ZnO heterostructures gives rise to an enhancement in the optical absorption compared with the parent monolayers.

Furthermore, in order to have a better understanding the band alignment in SiS/GeC and SiS/ZnO heterostructures, we plot the projected density of states (PDOS) of all atoms. We find that both the VBM and CBM of SiS/GeC heterostructure at the *K* point are mainly contributed by the Ge-p_*z*_ and C-p_*z*_ orbitals in the GeC layer, as depicted in Fig. 4(c). This finding suggests that SiS/GeC heterostructure gives rise to the generation of type-I straddling gap band alignment. Thus, SiS/GeC heterostructure is suitable material for light emission applications with the ultra-fast recombination between electrons and holes. In the other hand, the electronic states of S-p_*z*_ orbitals of the SiS layer contribute to the CBM of the SiS/ZnO heterostructure, while the VBM is dominated by the Zn-p_*y*_ state of the ZnO layers, as depicted in Fig. 4(d). It is obvious that the CBM and VBM are located in different constituents layers of SiS/ZnO heterostructure, therefore a clear type-II band alignment (staggered gap) has been exhibited by SiS/ZnO heterostructure, which can efficiently separate photogenerated electron–hole pairs and extend the lifetimes of carrier. The type-II band alignment is most desirable for solar energy conversion and photocatalytic activity.

**Fig. 5:**
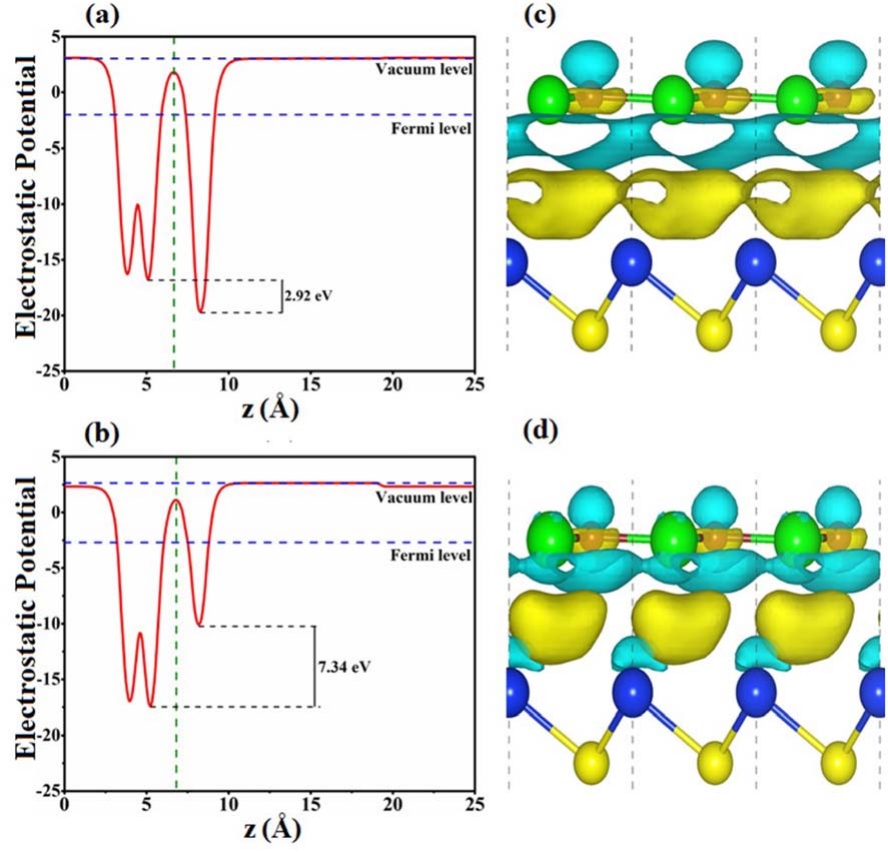
Electrostatic potentials of (a) SiS/GeC and (b) SiS/ZnO heterostructures along the *z* direction. Charge density difference of (c) SiS/GeC and (d) SiS/ZnO heterostructures. The charge accumulation and depletion are represented by yellow and cyan colors, respectively.

The interfacial features of SiS/GeC and SiS/ZnO heterostructures are explored by calculating the electrostatic potentials and charge density differences, as illustrated in Fig. 5. At the interfacial region of the SiS/GeC and SiS/ZnO heterostructures, the charge redistribution will be caused by the difference in work functions between the constituent monolayers. The calculated work functions of SiS/GeC and SiS/ZnO heterostructures are 4.354 eV and 5.356 eV, respectively. The difference in the work functions of the monolayers leads to the formation of a potential drop at the heterostructure interface. The potential drop in SiS/GeC and SiS/ZnO heterostructures are calculated to be 2.92 and 7.34 eV, respectively. Interestingly, the SiS layer has a higher potential than the GeC layer in the SiS/GeC heterostructure but it has a lower potential than the ZnO layer in the SiS/ZnO heterostructure. To further understand the redistribution of charges in the SiS/GeC and SiS/ZnO heterostructures, a Bader charge population analysis^45^ and charge density difference are calculated as below:

4 Δ*ρ* = *ρ*_H_ − *ρ*_M1_ − *ρ*_M2_

Here, *ρ*_H_, *ρ*_M1_ and *ρ*_M2_ denote charge density of heterostructures, the isolated SiS and GeC (ZnO) monolayers, respectively. The negative and positive charges denote by the cyan and yellow regions, respectively, as depicted in Fig. 5(c) and (d) for SiS/GeC and SiS/ZnO heterostructures. One can find that the electrons are mostly depleted in the upper layer of GeC layers in SiS/GeC heterostructure and Zn layer in SiS/ZnO heterostructure. Whereas, they are accumulated at the interlayer region for SiC layer. Bader population analysis shows that there is a small amount of charge of about 0.023*e* (0.007*e*) are transferred from SiS to GeC (ZnO) monolayer, leading to formation of a built-in electric field at the interface, which can efficiently separate the photogenerated charge carriers. This finding results in p-doping of the SiS layer and n-doping of the GeC and ZnO layers.

**Fig. 6:**
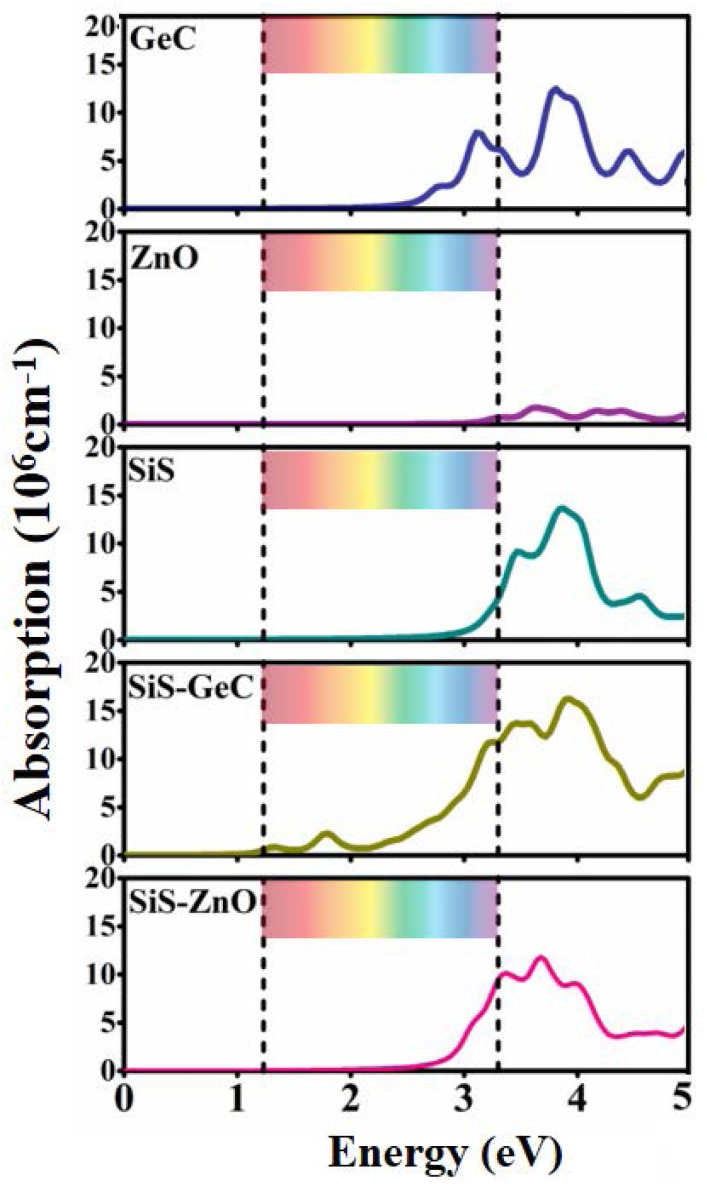
The light absorption spectra of GeC, ZnO, SiS monolayers, SiS/GeC and SiS/ZnO heterostructures. The ribbon region represents the range of visible light.

Next, we explore the optical characteristics of the SiS/GeC and SiS/ZnO heterostructures as well as the constituent monolayers. Optical properties are investigated by optical absorption spectra as a function of photon energy, as depicted in Fig. 6. Both the SiS/GeC and SiS/ZnO heterostructures exhibit good absorption in the visible and ultra-violet part of the spectrum. The reason is due to interlayer coupling and charge transfer, which can cause electronic states in the valence bands of the heterostructure to overlap, thereby increasing optical absorption. Additionally, the absorption spectra for SiS/GeC and SiS/ZnO heterostructures demonstrate that they have a wide visible light absorption region with an intensity of 10^6^ cm^−1^. Remarkably, the better visible performance of GeC monolayer leads to the excellent visible performance of SiS/GeC heterostructure and the absorption spectra of SiS/ZnO heterostructure has been enhanced in the visible region, despite the poor visible performance of SiS and ZnO monolayers. The SiS/GeC and SiS/ZnO heterostructures show a lower band gap than the corresponding monolayers, therefore a red-shift in the ultraviolet light region is evidently observed. Since SiS/GeC and SiS/ZnO possess modulation ability of excitation wavelength from ultraviolet to near-infrared region, Therefore, it is expected that both the SiS/GeC and SiS/ZnO heterostructures are promising for light harvesting and solar cells applications and other photoenergy conversion processes in the future.

**Fig. 7:**
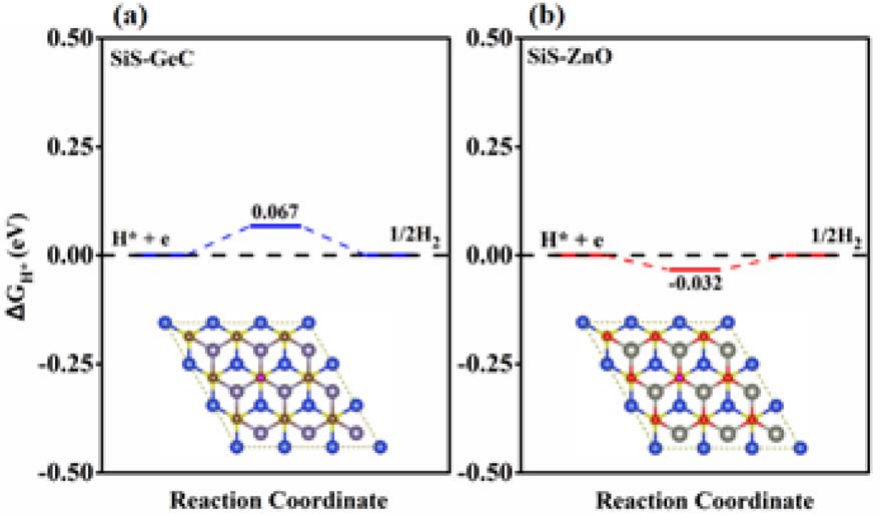
Calculated free energy (Δ*G*_H*_) diagram of HER at the equilibrium potential (*U*_RHE_ = 0 V) for the (a) SiS/GeC and (b) SiS/ZnO heterostructures.

Technology-wise, the HER is essential for creating hydrogen from water as a renewable energy source. Due to the wide variety of 2D material combinations that can be used, two-dimensional heterostructures in particular are one of the most promising HER approaches. Accordingly, we further explore the catalytic activity for the HER by investigating the Hydrogen (H) adsorption properties of the SiS/GeC and SiS/ZnO heterostructures. In this case possible adsorption sites were considered. Fig. 7 exhibits the optimized adsorption configurations of a H atom at the C and O sites of SiS/GeC and SiS/ZnO heterostructures, respectively. The H atom can be adsorbed on top of Ge, C, Zn and O atoms, whereas no adsorption configurations were stable on the bridge and hollow sites. Accordingly, we refer to the C and O adsorption configurations and calculated Δ*E*_H*_ with respect to the site at the C and O atoms in SiS/GeC and SiS/ZnO heterostructures, respectively. Adsorption energies (Δ*E*_H*_) and Gibbs free energy of hydrogen adsorption (Δ*G*_H*_) for both SiS/GeC and SiS/ZnO heterostructures are −3.55 (−3.67) eV and −0.067 (−0.032) eV for C (O) adsorption site, respectively. The HER was explored to study the performance of SiS/GeC and SiS/ZnO heterostructures for photocatalytic water splitting. There are two principal steps in HER catalysis. The first one is the Volmer reaction which is referred to as electrochemical hydrogen adsorption (H^+^ + e^−^ → H*), where H* denotes an adsorbed H atom. The second stage is the process of desorption, called the Heyrovsky reaction (H* + H^+^ + e → H_2_) or the Tafel reaction (H* + H* → H_2_). To promote the desorption reaction, a moderate Δ*E*_H*_ is desired for the HER. Within this context, the C and O sites show a balanced value of Δ*E*_H*_ to be −3.55 and −3.67 eV for both SiS/GeC and SiS/ZnO heterostructures, respectively.

The absolute value of Gibbs free energy of hydrogen adsorption (Δ*G*_H*_) is a widely used descriptor and reversely proportional to the catalytic activity for HER.^46,47^ Particularly, zero free energy is preferred for hydrogen evolution reaction performance. The formation of both SiS/GeC and SiS/ZnO heterostructures reduces Δ*G*_H*_ significantly, and the values for the C and O sites become −0.067 eV and −0.032 eV, respectively, which are very close to the best criteria.^48^ These data suggest that the SiS/GeC and SiS/ZnO heterostructures can exhibit a much better activity for HER than corresponding individual monolayers, as depicted in the free energy diagrams of HER over SiS/GeC and SiS/ZnO heterostructures and the corresponding models for the calculation.

## 4 Conclusions

In summary, first-principles calculations are preformed to investigate the electronic structure, optical properties and photocatalytic performance of the SiS/GeC and SiS/ZnO heterostructures. Our results demonstrate that both the SiS/GeC and SiS/ZnO heterostructures are energetically and thermodynamically stable at room temperature. We found that SiS/GeC heterostructure possesses a direct band gap semiconductors, whereas the SiS/ZnO heterostructure exhibits an indirect band gap semiconductor. Furthermore, the generation of the SiS/GeC and SiS/ZnO heterostructures gives rise to the reduction in the band gap as compared to the constituent monolayers, suggesting that they will have an enhancement in the optical absorption. Interestingly, the SiS/GeC heterostructure forms type-I straddling gap band alignment, while the SiS/ZnO heterostructure generates type-II staggered gap one. Absorption spectra reveal that considerable light absorption is observed in the visible and ultraviolet regions. Furthermore, SiS/GeC and SiS/ZnO heterostructures exhibit a red-shift, which further improve the efficient separation of photogenerated charge carriers. Both the SiS/GeC and SiS/ZnO heterostructures have Gibbs Free energy Δ*G*_H*_ very close to zero and are consider optimal to perform hydrogen evolution reaction, thereby suggesting that the these two heterostructures can show a much better activity for HER than corresponding individual monolayers. Consequently, the current SiS/GeC and SiS/ZnO heterostructures are considered promising for photovoltaic cells and efficient photocatalysts.

## Conflicts of interest

There are no conflicts to declare.

## Supplementary Material
