# Correction: Theoretical prediction of the electronic structure, optical properties and photocatalytic performance of type-I SiS/GeC and type-II SiS/ZnO heterostructures

**DOI:** 10.1039/d3ra90032k

**Published:** 2023-04-06

**Authors:** S. S. Sabir, H. U. Din, Sheraz Ahmad, Q. Alam, S. Sardar, B. Amin, M. Farooq, Cuong Q. Nguyen, Chuong V. Nguyen

**Affiliations:** a Department of Physics, Hazara University Mansehra KP Pakistan farooq@hu.edu.pk; b Computational Science Research Center, Korea Institute of Science and Technology (KIST) Seoul 02792 Republic of Korea 025264@kist.re.kr; c Department of Physics, Bacha Khan University Charsadda KP Pakistan haleem.uddin@yahoo.com; d School of Materials Science and Engineering, Institute of New Energy Material Chemistry, Nankai University Tianjin 300350 P. R. China; e Department of Physics, Abbottabad University of Science & Technology Havelian Abbottabad KP Pakistan; f Institute of Research and Development, Duy Tan University Da Nang 550000 Vietnam nguyenquangcuong3@duytan.edu.vn; g Faculty of Natural Sciences, Duy Tan University Da Nang 550000 Vietnam; h Department of Materials Science and Engineering, Le Quy Don Technical University Hanoi Vietnam

## Abstract

Correction for ‘Theoretical prediction of the electronic structure, optical properties and photocatalytic performance of type-I SiS/GeC and type-II SiS/ZnO heterostructures’ by S. S. Sabir *et al.*, *RSC Adv.*, 2023, **13**, 7436–7442, https://doi.org/10.1039/d3ra01061a.

The authors regret that the name of the first author (Shah Saleemullah Sabir) was shown incorrectly in the original article. The corrected author list is as shown above.

The Royal Society of Chemistry apologises for these errors and any consequent inconvenience to authors and readers.

## Supplementary Material

